# Wafer-scale nanofabrication of sub-5 nm gaps in plasmonic metasurfaces

**DOI:** 10.1515/nanoph-2024-0343

**Published:** 2024-08-28

**Authors:** Jeetendra Gour, Sebastian Beer, Pallabi Paul, Alessandro Alberucci, Michael Steinert, Adriana Szeghalmi, Thomas Siefke, Ulf Peschel, Stefan Nolte, Uwe Detlef Zeitner

**Affiliations:** Friedrich Schiller University Jena, Faculty of Physics and Astronomy, Abbe Center of Photonics, Institute of Applied Physics, Albert-Einstein-Str. 15, 07745 Jena, Germany; Fraunhofer Institute for Applied Optics and Precision Engineering IOF, Albert-Einstein-Str. 7, 07745 Jena, Germany; Faculty of Physics and Astronomy, Friedrich Schiller University Jena, Institute of Solid State Theory and Optics, Max-Wien-Platz 1, 07743 Jena, Germany; HM Hochschule München University of Applied Sciences, Department of Applied Sciences and Mechatronics, Loristr. 19, 80335 Munich, Germany

**Keywords:** plasmonics, nanogap, ALD, nanotechnology, nanogap metasurfaces, sub-5 nm

## Abstract

In the rapidly evolving field of plasmonic metasurfaces, achieving homogeneous, reliable, and reproducible fabrication of sub-5 nm dielectric nanogaps is a significant challenge. This article presents an advanced fabrication technology that addresses this issue, capable of realizing uniform and reliable vertical nanogap metasurfaces on a whole wafer of 100 mm diameter. By leveraging fast patterning techniques, such as variable-shaped and character projection electron beam lithography (EBL), along with atomic layer deposition (ALD) for defining a few nanometer gaps with sub-nanometer precision, we have developed a flexible nanofabrication technology to achieve gaps as narrow as 2 nm in plasmonic nanoantennas. The quality of our structures is experimentally demonstrated by the observation of resonant localized and collective modes corresponding to the lattice, with Q-factors reaching up to 165. Our technological process opens up new and exciting opportunities to fabricate macroscopic devices harnessing the strong enhancement of light–matter interaction at the single nanometer scale.

## 1 Introduction

In plasmonics, the interaction between an electromagnetic field and free electrons in a metallic nanoantenna results in sub-wavelength confinement of light [1], [2] at the characteristic resonance called localized surface plasmon resonance (LSPR) [3], [4]. Plasmonics is employed in a wide range of applications from sensing [5], [6], [7] to nonlinear optics [8], [9], [10]. In addition to LSPRs, plasmonic metasurfaces, which are bidimensional lattices of metallic nanoantennas, exhibit another type of resonance known as collective resonances (CRs). CRs are collective excitations of the array [11], [12], physically stemming from the hybridization between the localized mode and the surface waves, excited when the Rayleigh–Wood anomaly (RA) condition in grating-like structures is satisfied [13], [14]. CRs have been reported to enhance sensing [15], [16], [17] and nonlinear optical effects [18], such as second- [19], [20], [21], third-harmonic [22] generation, and four-wave mixing [23]. In a broader view, the importance of CR is strictly related to the very recent introduction of nonlocal metasurfaces [24], [25], [26] where the excitation of surface waves propagating along the structures is, on one side, opening a new possibility for light manipulation [27], and, on the other side, maximizing the nonlinear effects thanks to the longer interaction length.

While these resonances have their own advantages, a further increase in the field concentration can be achieved when the light is squeezed through nanometer-wide gaps of dimensions three orders of magnitude below the Abbe limit. At this very short scale, new phenomena become relevant, such as optically-induced electron tunneling [28], [29], electron quantum confinement [30], nonlocal and quantum effects [31], [32], [33]. However, an exhaustive experimental investigation in this regime is currently missing due to the lack of highly reliable fabrication techniques for ultra-thin nanogaps below 5 nm width.

To recapitulate, the maximization of the light–matter interaction on bidimensional structures, and possibly the realization of optical devices upon such effects, requires the efficient and reliable realization of nanogaps of a few nanometers width over a wide surface. As a “side effect”, this endeavor automatically would lead to a new experimental platform based on meta-optics, where microscopic quantum effects directly determine the macroscopic response [31].

To address this challenge, various methods have been proposed [8], [34], [35], [36], [37]. One of the advanced approaches [38] utilizes electron beam lithography (EBL) operating at 200 kV coupled with a high-resolution resist, such as hydrogen silsesquioxane (HSQ) [38], [39], facilitates the realization of structures with widths down to 1 nm, achieved by minimizing the point-spread function associated with the EBL. However, the method is plagued by lack of reproducibility and uniformity at this scale. Isolated structures are even wider than such a limit, due to the mechanical stability of the HSQ [38]. In Ref. [38] the authors have employed an aberration-corrected scanning transmission electron microscope (STEM), which inherently limits the number of structures that can be fabricated, thereby imposing a significant constraint on scalability. Despite these challenges, EBL still offers the potential to achieve even Ångstrom-size gaps using the overlay technique, however at the expense of a low process yield [40]. Another way of writing nanogaps is based on focused Helium ion beam (He^+^-FIB) milling [41], [42], [43]: using a “Sketch and Peel” strategy capable of a drastic reduction in the fabrication time [44], dimers having nanogaps down to 5 nm have been demonstrated [45]. Another solution is nano-imprint lithography, where post-inscription physical and chemical processes improve the resolution of UV lithography [46]. The fabrication of sub-10 nm gaps in nanoantennas with sharp edges has been achieved using cascade domino lithography (CDL) [47], whose uniformity in fabricating arrays of ultra-sharp nanogapped antennas has been further improved with the so-called guided domino lithography (GDL) [48].

All the techniques described above allow arbitrary shaping of the optical antennas. Nonetheless, exploiting them it is extremely difficult to achieve gaps of a few nanometers in a controllable and efficient manner. On top of that, the problem of the scaling up over wide areas requested by plasmonic metasurfaces with high-throughput is, at least currently, even a greater challenge. Alternative approaches based on atomic layer deposition (ALD), called atomic layer lithography (ALL) [49], caught widespread attention due to the unique ability to fabricate nanogapped plasmonic structures down to single nanometer width, with sub-nanometer precision and being strongly homogeneous over wafer-scale. Basically, an ultra-thin layer of a sacrificial oxide grown by ALD-later removed via wet etching-establishes a nanogap between two sequentially deposited metal layers, regardless of their shape. However, current realizations of ALL method [49], [50], [51] do not allow the independent shaping of the nanogaps and of the nanoantenna, thus strongly limiting the type of feasible geometries. An exception is ref. [52], where gaps of minimum 5 nm are achieved by combining ALD with photolithography, the latter thus inhibiting the realization of optical antennas due to the low resolution.

In this Article, we introduce a reliable, flexible and robust nanofabrication technology to realize ultra-thin sub-5 nm plasmonic nanogap metasurfaces. After demonstrating the fabrication of nanogapped grating structures down to 2 nm width, we use these nanogapped gratings as a basis for the following fabrication of sub-5 nm nanogaps in metasurfaces made of gold bow-tie nanoantennas (BNAs). The metasurfaces with nanogaps of 2 nm and 3 nm width are finally characterized in the linear optical regime, discussing both the dependence of their response on the fabrication parameters and on the aging of these structures.

## 2 Results and discussion

Top-down fabrication of plasmonic nanostructures traditionally makes use of EBL, metal evaporation, and standard lift-off processes. It allows flexible design and high yield [53], but the achievable feature sizes are limited by the resolution of the electron-beam resist and other factors [38], [54]. We circumvent these intrinsic limitations using an approach based on a two-step EBL process [40], achieving metallic nanostructures separated by dielectric spacers below 5 nm, additionally encompassing a sub-nanometer precision in the definition of the gap. Although Zhu et al. [40] utilizes overlay alignment process to achieve Ångstrom-scale gap-widths, their technique suffers from an intrinsic low throughput, in turn inhibiting uniformity and scalability over wide areas. We address this issue by combining ALD with EBL to achieve a uniform gap thickness over a whole wafer.

**Figure 1:**
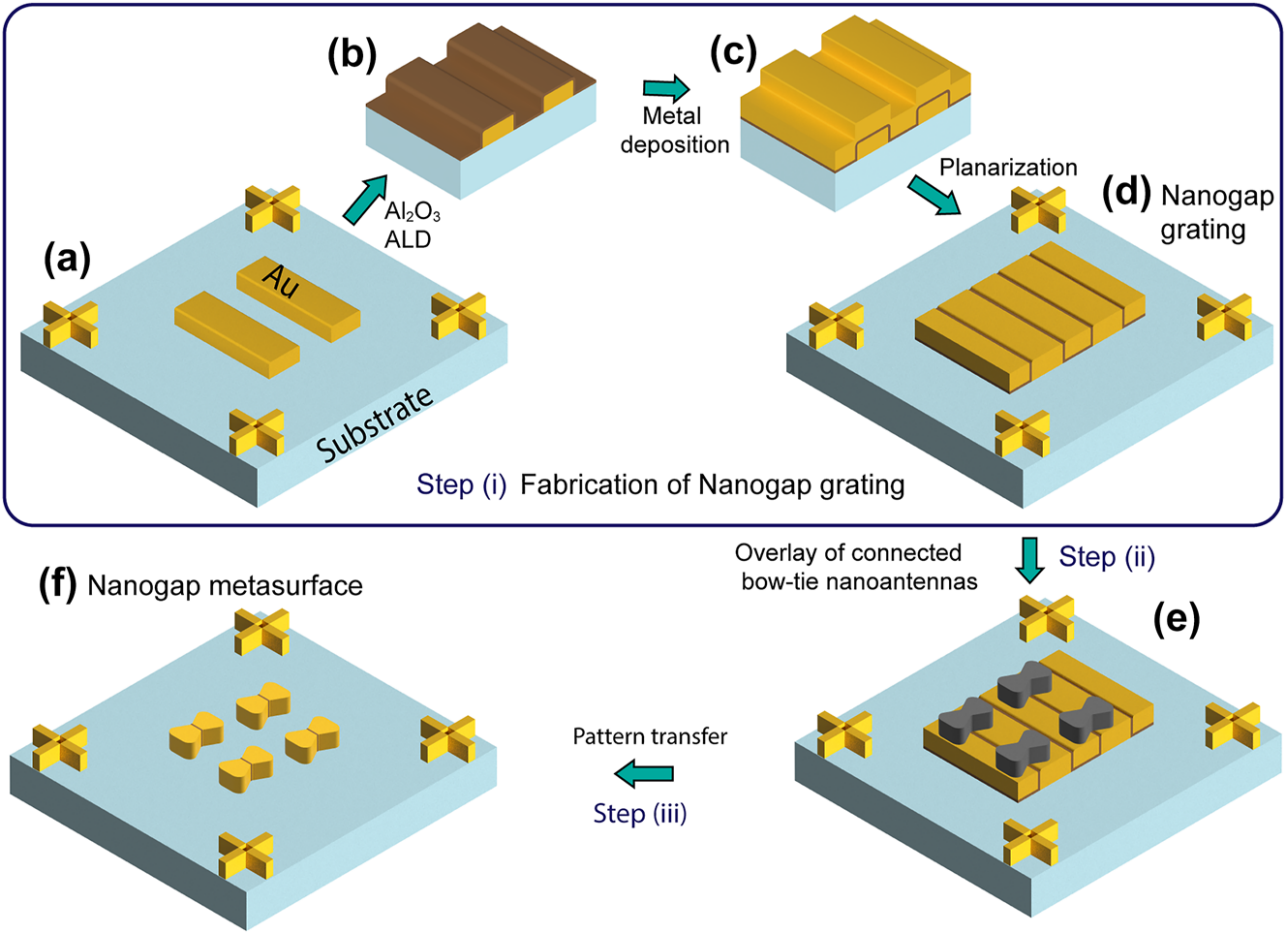
Fabrication process flow to create plasmonic nanostructures with sub-5 nm nanogaps. (a)–(d) Detailed steps for the realization of the nanogap gratings in step (i). (e) Overlay of the connected bow-tie nanoantennas using the alignment markers in the second EBL exposure in step (ii). (f) Realized nanogap metasurface after the pattern transfer process using ion beam etching in step (iii).

Before discussing our fabrication process, we highlight the design of the metasurface that we will fabricate; nonetheless, we stress out that our technique can be applied to any kind of nanoantenna shape, gap profiles, and lattice arrangements. With a focus on resonant field enhancement in nanogaps the sample was designed to promote both LSPRs and CRs of an array of BNAs. Both resonances were optimized for an excitation under normal incidence with an electric field polarized in the *x*-direction along the antenna axis. While the spectral position of the LSPR is defined by the shape of the individual BNAs, the CR was tuned to a wavelength around 800 nm. It was induced by coupling the nanoantennas to a TE-polarized mode propagating into the *y*-direction in the dielectric layer beneath the antennas. We fabricated arrays having a period of 800 nm both along the *x*- and *y*-directions. The unit cell of our periodic arrays consists of two BNAs made of gold and embedding in their center a sub-5 nm nanogap made of a dielectric material, specifically aluminum oxide Al_2_O_3_. One of the two BNAs in a unit cell is shifted by 50 nm in the +*x*-direction to break 400 nm periodicity and realize an effective periodicity of 800 nm, see design in Supplementary Info Figure S2. This allows for excitation of the CR under normal incidence around 800 nm wavelength. Each metasurface is fabricated on a 100 nm thick Al_2_O_3_ layer on a Si substrate. In the final fabrication step, the silicon (Si) substrate underneath the Al_2_O_3_ layer is etched away from the backside, so that a membrane with the structures spans over a 500 µm diameter hole in the Si wafer and is suspended in air, see Supplementary Info. This choice facilitates both the measurement of the optical response in transmission, as well as the presence of guided light resonance capable of enhancing the propagation of surface waves along the array plane *xy* [55]. However, this membrane is not necessary to fabricate sub-5 nm plasmonic metasurfaces. The BNA metasurfaces embedding nanogaps are realized in three main steps, see Figure 1: (i) fabrication of nanogap gratings; (ii) EBL patterning using overlay technology, the latter permitting an accurate alignment (error well below 10 nm) of the nanogaps with respect to the center of the BNAs; (iii) pattern transfer from the resist structured in step (ii) into the bottom nanogap grating layer. We now briefly discuss the three main steps listed above.

**Figure 2:**
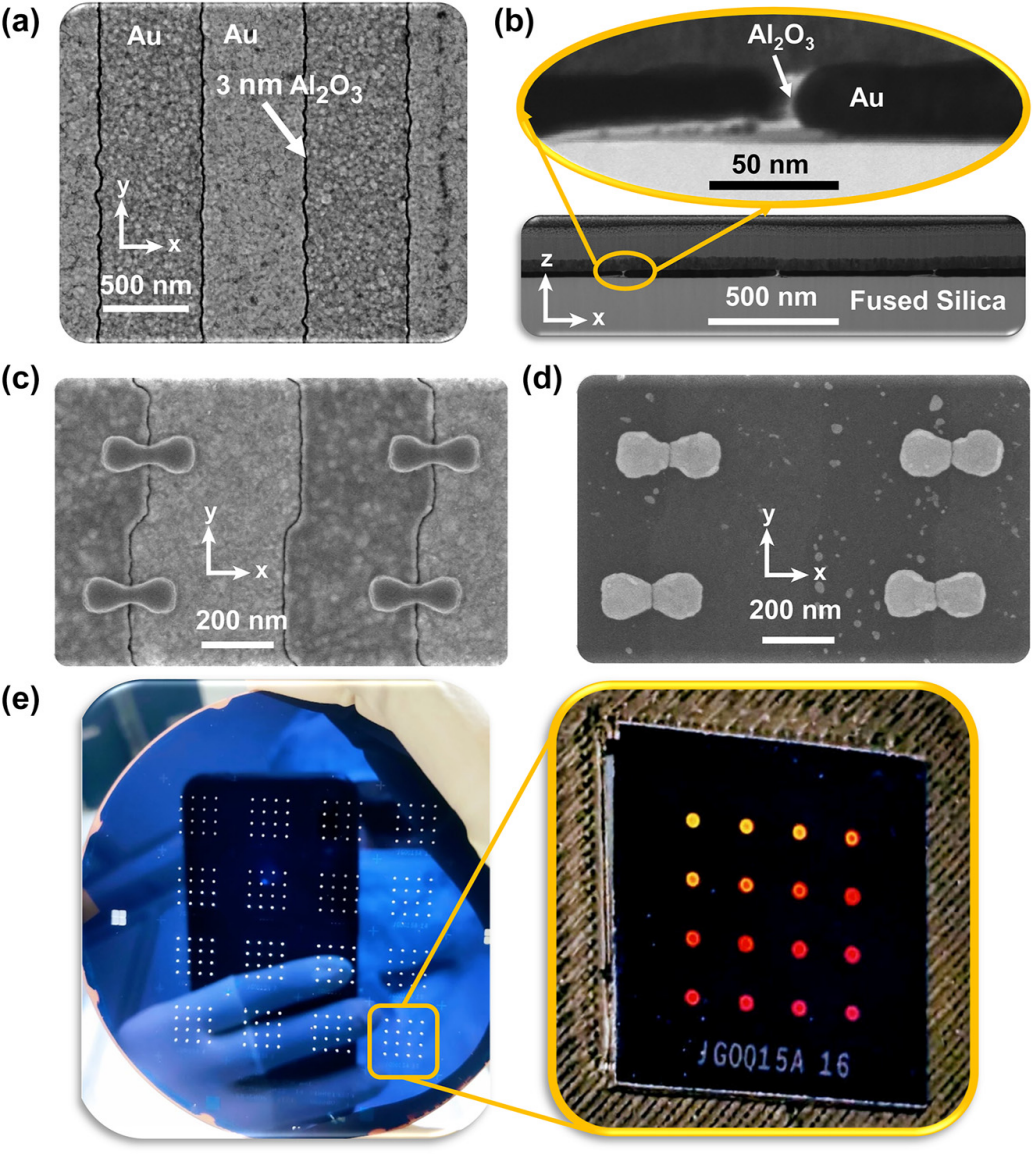
Scanning electron microscope images of sub-5 nm nanogap gratings and BNAs as fabricated. (a) 1D nanogap gratings having a period of *P*_*x*_ = 600 nm (b) cross-section view of the nanogap grating and high-resolution STEM image of 3 nm Al_2_O_3_ nanogap region in zoom. (c) Overlay of connected resist bow-tie antennas with center aligned to nanogap region and (d) 2 nm nanogap BNAs after pattern transfer. (e) Image of nanogap metasurfaces on a wafer of 100 mm diameter; the zooming shows a single chip of dimensions 15 mm × 15 mm.

In the first step (i), we fabricate vertically-oriented nanogap gratings using ALD at a growth rate of about 1.2 Å/cycle by following the process summarized in Figure 1(a)–(d). ALD, known for its precise thickness control at the sub-nanometer level, is used for creating conformal coatings [56], [57] of ultra-thin layers. The gap widths in our metasurfaces are determined by the thicknesses from ALD cycles. Initially, we measured these thicknesses on Si reference chips and later confirmed them via ellipsometry. Notably, ALD has been successfully utilized to develop nanogap gratings with features down to a single nanometer, as demonstrated by Chen et al. [50]. Considering the essential metal deposition step for nanogap formation, as shown in Figure 1(c), the method potentially allows the creation of nanogaps between different metals, despite the exclusive use of gold in this study. While the process is similar to Ref. [50], [51], improvements in the planarization process allow using thinner sacrificial metallic films, see Supplementary Info. Typical nanogratings, as viewed from the top (i.e., the plane *xy*) under scanning electron microscope, are shown in Figure 2(a). The top view of the nanogap grating shows the conformal nature of the ALD process as the nanogap lines, see black lines in Figure 2(a), follow the line-edge roughness profile of the initial grating, see Figure 1(b). A visualization of the nanogap cross-sectional profile is provided in Figure 2(b), where the inset in Figure 2(b) clearly depicts the Al_2_O_3_ dielectric spacer layer within the nanogap image obtained via scanning transmission electron microscope (STEM). Although STEM images clearly show the dielectric between the gold grating ridges, accurate gap size estimation is challenging due to the lamella’s finite thickness and sample homogeneity along the beam axis, the latter being limited by line edge roughness of the grating and the vertical gap-width variation across the gap region. During step (i) we also fabricate the alignment markers, later to be used for the overlay alignment process, see Figure 1(a). Note that the nanoslits are not rectilinear due to the fact that one unit cell contains two shifted BNAs, see Figure 2(c). This demonstrates the versatility in our approach in defining nanogaps of different shape and arrangement.

In the second step (ii), connected (i.e., no gaps) BNAs are patterned in a resist layer via EBL on the top of the nanogap gratings, with their centers aligned using the markers fabricated along with the nanogratings in step (i) [6], [58], see Figures 1(e) and 2(c). For achieving a high-throughput during the pattern writing the E-beam exposure was performed using both the variable shaped beam and the cell-projection mode [59] of the SB350 OS e-beam writer (VISTEC GmbH). Over the whole wafer, our process achieves typically an offset well within 25 nm from the original design [60]. This overlay alignment error can be caused by various factors, such as wafer distortion, process variations, quality of the alignment markers, and finally environmental fluctuations. Further improvements in alignment strategy can reduce overlay errors and bring the positional offset well below 10 nm [61]. To improve the nanogap positional offset, one should also reduce line-edge roughness (i.e. straightness of the grating edges after lift-off in the initial grating) and reduce overall alignment errors. It is important to notice that each chip contains different sets of alignment markers, thus yielding negligible variation of positional offset within each single chip. Nonetheless, as our method is based on ALD conformal coating the accuracy of achieved gap sizes is defined by ultra-thin uniform and conformal coatings from ALD and independent of the much larger alignment errors of tenths of nanometers.

In step (iii), see Figure 1(f), we finally obtain nanogapped plasmonic metasurfaces by a pattern transfer process using ion beam etching, see Figure 2(d). We can thus fabricate metasurfaces over wide areas containing uniform nanoantennas, see Figure 2(e) showing nanogap metasurfaces processed on a wafer of 100 mm diameter, each of them with very similar sub-5 nm nanogap. Previously, Chen et al. 2013 [50] and Yoo et al. 2016 [51] employed ALL to write nanogap arrays, but without the crucial possibility of shaping the profile of the antennas on *xy* plane independently, their proposal being indeed limited to uniform metallic thin films. In our work, we address the limitation by experimentally demonstrating independent shaping of the nanogap antennas over the wafer-scale, along with improvements with respect to the previous works in the planarization process permitting the realization of thinner metallic nanoantennas [50], [51]. Recently, an integrated multi-step fabrication approach based on photolithography, ALD, ion-milling along with overlay alignment enabled scalable fabrication of sub-10 nm gaps in split-ring resonators [52]. However, this approach is limited to terahertz (THz) devices as photolithography struggles to achieve features on the nanoscale, as required for optical antennas.

To summarize the technological part of our paper, we combined ALD, overlay techniques, variable shaped beam and character projection-based EBL patterning [59] to achieve a fast and efficient recipe to a wafer-scale sub-5 nm nanogap metasurfaces of arbitrary shape, emphasizing their scalability for the potential applications and photonic integration.

## 3 Linear optical characterization

**Figure 3:**
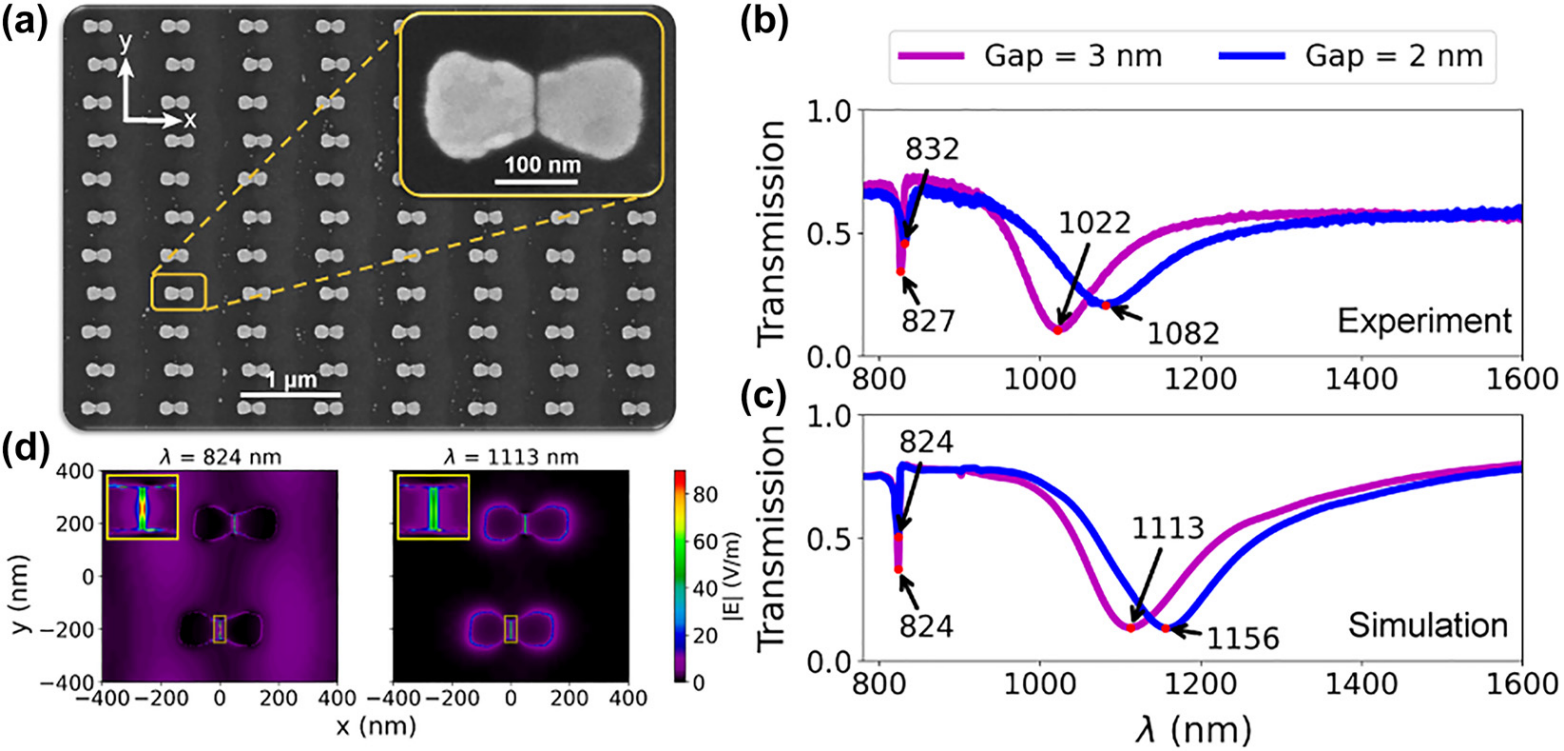
Experimental and simulated behavior of sub-5 nm nanogap metasurfaces. (a) Scanning electron microscope image for the case of 2 nm nanogap bow-tie. Inset: Zoom on a single antenna. (b) Measured transmission spectrum for 2 (blue curve) and 3 nm (violet curve) gaps. The corresponding numerical simulations are shown in panel (c). (d) Simulated distribution of the modulus of the optical field maps for a 3 nm nanogap metasurface at CR (824 nm) and LSPR (1,113 nm) positions. Inset: Magnification in the gap region. The incident field strength is 1 V/m. The optical field maps are sampled at the interface of the membrane and the nanoantennas.

In Figure 3(a), a scanning electron microscope (SEM) image of the top view of a 2 nm nanogap BNA metasurface is shown, where the black line at the center corresponds to the 2 nm nanogap. The transmission spectra of a 2 nm and a 3 nm gap metasurface measured in the 0th order and at normal incidence are shown in Figure 3(b). Hereafter, in all of our measurements and simulations the polarization of the electric field is parallel to the long axis of the BNAs, such choice corresponding to the maximum field enhancement. Experiments and simulations plotted in Figure 3(b) and (c) are in good agreement: the spectra encompass an LSPR as a broad dip and a CR as a narrow dip demonstrating Q-factors up to 165. With respect to plasmonic metasurfaces in the presence of collective resonances, the achieved Q-factors are of the same order of magnitude with respect to the state-of-the-art [62], [63], [64], but in these papers the nano-antennas do not encompass any nanogap below 5 nm. Numerical simulations predicted Q-factors around 10^3^ both for monomer and dimer antennas [65], [66]; such Q-factors have been measured recently for SLR in monomers shaped as nanobars [67], but the overall field enhancement is half than our due to the absence of a nanogap. The slight differences in the CR resonance wavelengths, consisting in deviations of ≈2 nm and ≈7 nm between measured and simulated response, can be ascribed to several potential sources. These include small inhomogeneities during membrane back etching, nonuniformities in thick ALD layers (approximately 3 % across entire reactor), EBL patterning effects, and residual stress in the membrane. While each factor can influence the CR resonance wavelength in a way, pinpointing a single dominant cause remains challenging. In Supplementary Info, we present simulation results demonstrating the impact of small deviations from the designed values of the membrane thickness and of the period along the *x*-direction, see Supplementary Info Figure S10.

According to the simulations (see Figure 3(d) for a gap of 3 nm), the optical field is greatly enhanced nearby the CR, more than at the LSPR, although the enhancement is of the same order of magnitude. Furthermore, the field expands all over the metasurface owing to the excitation of a surface wave. The presence of the membrane featuring a large refractive index greatly helps the excitation of surface waves, thus boosting up the Q factor of the CRs [55].

Whereas Figure 3 shows the response of a typical sample, Table 1 summarizes the statistical response from the several samples over the wafer and its comparison with the simulation of ideal structures. Qualitatively speaking, the gap size plays a dominant role in determining LSPR position. Table 1 shows significant quantitative differences for LSPR; the CR, which is mainly determined by the overall periodicity, it is well reproduced both in shape and in position. However, for LSPRs, the general trend is reproduced, i.e., narrower gaps induce a red-shift. As a matter of fact, the smaller the gap, the stronger the coupling and the more the optical response is dominated by just one large particle instead of two small ones, and bigger particles are known to have an LSPR at longer wavelengths. But, the amount of red-shift differs between experiments and simulations significantly, which hints to a lack of understanding with respect to gap formulation and optical response at atomic scales. For nanogaps of 2 nm and 3 nm, the simulated LSPR positions are red-shifted compared with the experimental ones by approximately 75 nm (±8 nm) and 86 nm (±5 nm), respectively. The measured LSPR position is red-shifted by about 54 ± 9 nm when the gap size is reduced from 3 nm to 2 nm, whereas a shift of about 43 nm is predicted by the numerical simulations, see Table 1. The discrepancy between simulation and experimental results might have several reasons. First, the gap profile is not totally straight along the *xy* plane due to the polycrystalline nature of the deposited gold, moreover the gap width varies along the vertical direction *z*, as shown in Figure 2(b). Those imperfections with respect to the ideal design are not accounted for in the FDTD simulations. Second, purely classical models of the electron gas in the gold particles interacting via the gap fail as a local response with a well-defined dielectric constant becomes questionable for atomic dimensions. For gaps below 5 nm one expects the emergence of quantum effects in proximity of the metal interface, including phenomena such as nonlocal effects, electrons spill-out, Landau damping [31], [40]. Such effects can be accounted for using e.g. the so-called Feibelman parameters, yielding the introduction of a new set of boundary conditions at metallic interfaces to be inserted in the classical Maxwell equations [31]. In the presence of nanogaps of 10 nm and lower, Yang et al. 2019 [31] observed a discrepancy between experiments and classical model based upon the local response approximation and infinitely sharp interfaces, the difference consisting in a blue-shift of an LSPR for a disk separated from a metallic substrate by a nanogap. Accordingly, we also observed a blue-shift between the measured and simulated LSPR in our sub-5 nm nanogap metasurfaces (see Figure 3(b) and (c) and Table 1). Nonetheless, a complete discussion of these nonlocal effects in our metasurfaces is outside the scope of the current work and is left to the following and more technical works.

**Table 1:** LSPR and CR positions for the experimental (measured average with standard deviation from multiple chips, six for 2 nm gap case and three for 3 nm gap case, with the same metasurface dimensions) and simulated 0th order transmission spectrum.

Gap (nm)	Experimental		Simulation	
	*λ*_*LSPR*_ (nm)	*λ*_*CR*_ (nm)	*λ*_*LSPR*_ (nm)	*λ*_*CR*_ (nm)
2	1,080.7 ± 8.0	830.3 ± 2.1	1,156.1	823.9
3	1,026.3 ± 4.8	825.3 ± 2.5	1,112.7	823.9

**Figure 4:**
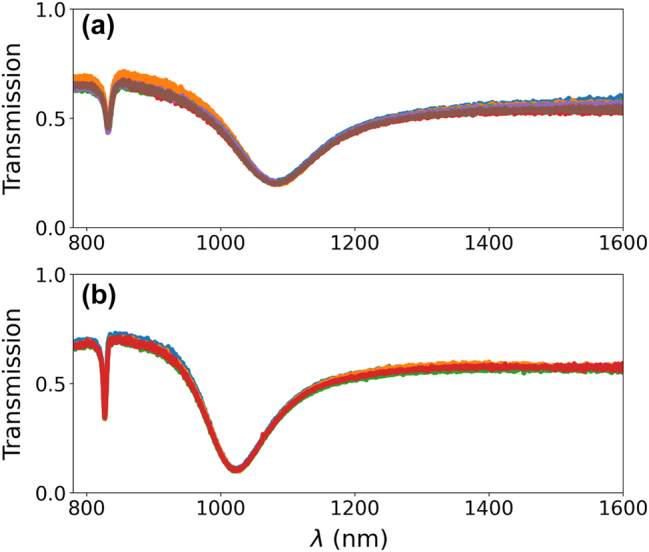
Uniformity of the optical response in the linear regime. Measured transmission spectra at different probe beam positions on the metasurface for the case of a (a) 2 nm gap and a (b) 3 nm gap. Each curve corresponds to different spots (six in (a), four in (b)) on the metastructure.

Additionally, we tested the homogeneity of the metasurface by shifting the probe beam having 250 µm diameter along the plane *xy* but always ensuring a complete spatial overlap with the array (measurements for narrower beams are depicted in the Supplementary Info Figure S7). The response curves plotted in Figure 4(a) and (b) overlap almost perfectly. Our experimental findings demonstrate the successful fabrication of plasmonic nanogap metasurfaces. This achievement is particularly notable given the uniformity and homogeneity we observed across wafer-scale samples. Furthermore, the results from multiple metasurfaces further underscore the homogeneity and robustness of our fabrication process, as shown in Supplementary Info Figures S6–S9, which also highlights the influence of the gap length, width, and BNA dimensions on LSPR indicating how subtle dimensional adjustments are in tuning LSPR properties. Our preliminary analysis indicates that, while deliberate tuning of BNA gap lengths can modulate the LSPR response, comprehensive experimental and theoretical work is necessary to harness these effects for practical applications.

**Figure 5:**
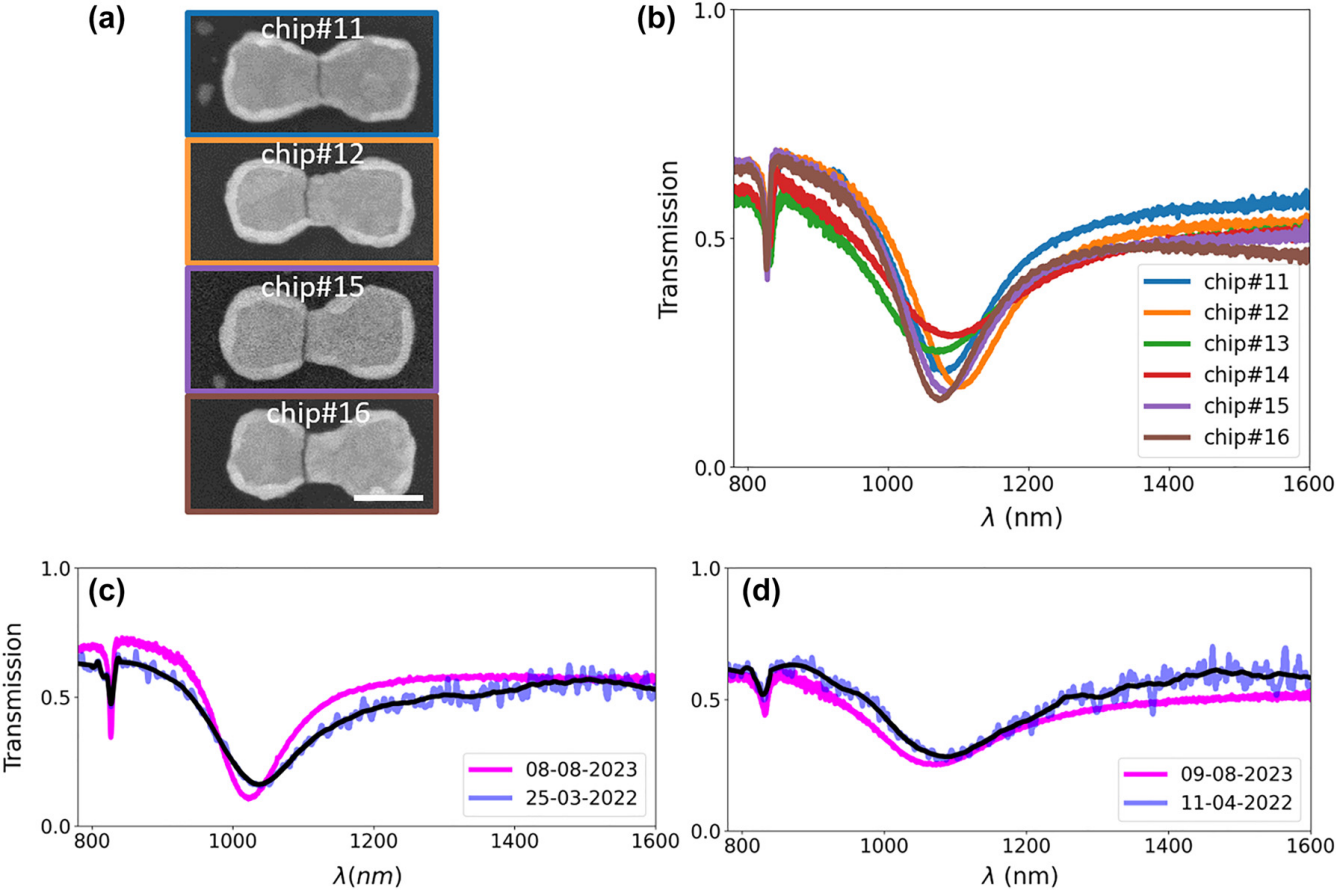
Influence of nanogap position and aging of the metasurfaces. (a) Scanning electron microscope images from different chips for a 2 nm gap; the scale bar is 100 nm long. (b) Acquired transmission spectra for different positions of the nanogap. Change in the optical transmission for (c) 2 nm and (d) 3 nm nanogaps with time. Noisy measured data (blue color with applied transparency) and fitted data (in black color) after applying savgol filtering.

We have also investigated how the relative position of the nanogap with respect to the antenna affects the optical response. In Figure 5(a), we show scanning electron microscope images of bow-tie antennas for the metasurface taken from different chips for 2 nm gap width. As discussed above, the EBL process provides a small misalignment across the wafer, which is nonetheless fixed within each chip. According to Figure 5(b), the CR remains the same within a very good accuracy (standard deviation of about 2 nm), while the LSPR position and shapes vary more significantly. Overall, the LSPR over the different chips fabricated on the 100 mm wafer is consistent with a resonance wavelength of 1,080.7 nm with a standard deviation of 8.0 nm for 2 nm gap case, thus avoiding any overlap with the response measured for the 3 nm gap case that show LSPR at 1,026.3 nm with a standard deviation of 4.8 nm. According to the numerical simulations (see Supplementary Info Figure S11), these slight deviations are mainly due to the different positions of the nanogap inside the plasmonic antennas (within ±25 nm). While overlay alignment affects LSPR, it does not limit the minimum gap sizes achievable. The variation in LSPR position can also be attributed to the nanoantenna thickness variation (below 5 nm) across the wafer. Consequently, slight variations in LSPR positions and optical response come from nanogap position offsets and small thickness variations of BNA nanoantenna across the wafer-scale, see Supplementary Info Figure S11. However, significant gap size variations are unlikely due to the highly conformal nature of ALD [68], [69], [70] and nonuniformities being below 3 % across the entire 200 mm ALD reactor, see Materials and Methods. Indeed, roughly 30 nm variations of the resonance caused by a displacement of the gap position by several tenths of nanometers, see Figure 5(a), are by far smaller than the 54 nm resonance shift induced by an increase of the gap width by a single nanometer. This again emphasizes the importance of a better understanding of the structure and response of the nanogap.

We finally studied the aging of these metasurfaces, a fundamental point when dealing with nanotechnology in general. Measurements 17 months after the fabrication show a slight blue-shift compared to the measurements performed just after the sample fabrication, see Figure 5(c) and (d). In Table 2 the changes of the LSPR and CR wavelengths are reported, including their standard deviations obtained by statistical sampling over different chips and the metasurface contained in each chip. The changes over time in the locations of the LSPR are of similar magnitude for both nanogap thicknesses. The blue shift in LSPR position suggests a slight increase of the nanogap size. These changes may arise from a number of factors such as gold diffusion across the oxide, membrane stress, and ambient environmental conditions. Further investigations to evince the responsible effects will be conducted in the future.

**Table 2:** Measured LSPR and CR positions after 17 months.

Time	Gap = 3 nm		Gap = 2 nm	
	*λ*_*LSPR*_ (nm)	*λ*_*CR*_ (nm)	*λ*_*LSPR*_ (nm)	*λ*_*CR*_ (nm)
2022	1,039.9 ± 4.1	826.7 ± 0.6	1,092.7 ± 6.0	829.6 ± 0.8
2023	1,026.3 ± 4.8	825.3 ± 2.5	1,080.7 ± 8.0	830.3 ± 2.1

## 4 Conclusions

In summary, we presented an advanced, robust and scalable fabrication technology capable of producing wafer-scale plasmonic metasurfaces having bow-tie nanoantennas embedded with vertically oriented nanogaps as narrow as 2 nm with sub-nanometer accuracy by utilizing atomic layer deposition along with high-throughput electron beam lithography techniques, enabling the flexibility of nanogap patterning in nanoantennas of arbitrary shapes.

Apart from the visualization of the nanostructures with electron microscopy, the optical response of these metasurfaces in the linear regime shows a strong dependence on the localized resonance affected by the gap size. Even more importantly, the transmission spectrum confirms the high degree of homogeneity of the metasurfaces by showing collective resonances with Q-factors up to 165. The transmission spectra are subject only to tiny variations when different regions of the arrays are probed. With respect to future perspectives, sub-5 nm nano-gap metasurfaces can be of pivotal relevance as an effective bridge between nano-emitters of various nature and their use in real-world applications [66], [71], [72]. On a more physical point of view, these structures are a relevant step forward towards the reproducible fabrication of metasurfaces with dimensions potentially down to atomic scale, for which light–matter interaction significantly relies on quantum effects.

## 5 Materials and methods

### 5.1 Atomic layer deposition

In order to grow Al_2_O_3_ thin films by thermal atomic layer deposition (ALD), we used trimethyl aluminium (TMA) as metal-organic precursor and water (H_2_O) as oxidizing agent, respectively. To fabricate thin membranes, we deposited a 100 nm thick Al_2_O_3_ thin film on a cleaned 100 mm diameter Si wafer using a SunALE R-200 (Picosun Oy, Masala, Finland) reactor. The depositions were done at 350 °C to reduce residual film stress and to avoid wrinkling of the membrane after the fabrication. The optimized ALD process parameters are as follows: 0.1s TMA pulse, 4s N_2_ purge, 0.2s H_2_O pulse, and 4s N_2_ purge obtaining a growth per cycle of about 0.9 Å/cycle [57]. By ellipsometric measurements (M-2000, J.A. Woollam Co.) of the thickness of films deposited on a reference Si wafer, we found the thickness to be approximately 102 nm, with the non-uniformity across the entire ALD reactor (200 mm diameter) below 3 %. The RMS surface roughness of these coatings measured by an AFM over a scan area of 1 µm × 1 µm is about 0.3 nm [73], [74].

For the conformal coatings over the grating structures, we grew ultra-thin Al_2_O_3_ films by means of plasma-enhanced atomic layer deposition (PEALD) technique using an Oxford Instruments (Bristol, United Kingdom) OpAL™ ALD reactor at 150 °C, equipped with an inductively coupled plasma, RF generator operating at 13.56 MHz. The optimized PEALD sequence is as follows: 0.03 s TMA pulse, 5 s Ar purge, 5 s O_2_ plasma (300 W, 50 sscm) exposure and finally 2.5 s of Ar purging of chamber leading to a growth per cycle (GPC) of about 1.2 Å/cycle [74], [75]. While ellipsometric measurements allow us to measure thin film thicknesses, characterizing ultra-thin films such as 2 nm and 3 nm remains challenging due to the uncertainties from the potential factors such as substrate effects, fitting models, instrumental limitations, and physical effects at the single nanometer scale. Nevertheless, our ellipsometric measurements yield uncertainties well within 0.5 nm.

### 5.2 Scanning transmission electron microscopy (STEM)

Scanning transmission electron microscopy (STEM) images of the nanogap region were obtained using FEI Helios NanoLab G3 UC operated at 30 kV with 25 pA current and 10 µs dwell time. The TEM lamella preparation was performed at an FEI Helios NanoLab G3 UC. At first the surface was protected with a localized layer of platinum using electron beam induced chemical vapor deposition (CVD). This was followed by *Ga* ^+^ ion beam induced CVD. A 1.5 µm thick lamella was then cut out using a 30 keV focused Ga ion beam and sputter welded at both ends to a copper TEM lift-out grid. Final thinning was performed using 30 keV Ga ions at 80 pA to a thickness of less than 100 nm.

### 5.3 FDTD simulations

All simulations were performed using finite difference time-domain (FDTD) calculations based on the commercial software Ansys Lumerical. To simulate the response of large periodic arrays, we applied periodic boundary conditions in the *x* and *y*-directions, whereas perfectly matched layers along the *z*-direction were implemented. The mesh override region was applied over the bow-tie antennas and separate mesh override was used for the nanogap region to get a finer resolution of one tenth of the nanogap size. For the optical constants of gold used in the simulations, we took the material data from Ref. [76]. The optical constants for ALD-grown Al_2_O_3_ were found by ellipsometric characterization of the uniform thin films deposited. For the unit-cell, we directly imported the SEM images of the plasmonic nanoantennas into the software, thus, simulating structure as similar as possible to the fabricated ones.

## Supporting Information Available

The Supporting Information is available free of charge at https://doi.org/10.1515/nanoph-2024-0343.

## Supporting Information

Additional sample fabrication information: Wafer-scale sample design (Figure S1), bow-tie nanogap metasurface design (Figure S2), some of BNA parameters (Table 1) after fabrication, planarization process (S3–S4), thin film membrane fabrication. Linear optical characterization: optical setup (Figure S5), demonstrating homogeneity of fabrication (Figures S6 and S7), impact of dose factor and gap length of BNA on linear response (Figures S8 and S9), Q-factor calculation (Table 2). FDTD simulations of linear response and electric field maps: effect of small variations in period and membrane thickness (Figure S10), nanogap positional offset and BNA thickness effects (Figure S11), and, optical field maps of 2 nm gap (Figure S12).

## Supplementary Material

Supplementary Material Details
