# Supplementary material for: Wafer-scale nanofabrication of sub-5 nm gaps in plasmonic metasurfaces
Source: Nanophotonics. 2024 Aug 28;13(22):4191–202. doi: 10.1515/nanoph-2024-0343 (PMC11501063; doi:10.1515/nanoph-2024-0343)
Supplement: Supplementary file 1 — Supplementary Material Details [file j_nanoph-2024-0343_suppl_001.pdf]

Supporting Information

Wafer-scale nanofabrication of sub-5 nm gaps in plasmonic metasurfaces

Jeetendra Gour[1,*], Sebastian Beer[1,*], Pallabi Paul[1], Alessandro Alberucci[1], Michael Steinert[1], Adriana Szeghalmi[3], Thomas Siefke[1,3], Ulf Peschel[2], Stefan Nolte[1,3], Uwe Detlef Zeitner[1,3,4]

[1]Friedrich Schiller University Jena, Faculty of Physics and Astronomy, Institute of Applied Physics, Abbe Center of Photonics, Albert-Einstein-Str. 15, 07745 Jena, Germany, E-mail: jeetendra.gour@uni-jena.de

[2]Friedrich Schiller University Jena, Faculty of Physics and Astronomy, Institute of Solid State Theory and Optics, Max-Wien-Platz 1, 07743 Jena, Germany

[3]Fraunhofer Institute for Applied Optics and Precision Engineering IOF, Albert-Einstein-Str. 7, 07745 Jena, Germany

[4]HM Hochschule München University of Applied Sciences, Department of Applied Sciences and Mechatronics, Loristr. 19, 80335 Munich, Germany

[*]Corresponding author: jeetendra.gour@uni-jena.de; s.beer@uni-jena.de

S1 Sample Fabrication **3**

S1.1 Wafer Layout 3

S1.2 Bow-tie Metasurface Design 4

S1.3 Planarization Process 4

S1.4 Thin Film Membrane Fabrication 6

S2 Linear Characterization **6**

S2.1 Linear Transmission Setup 6

S2.2 Homogeneity of Fabrication 7

S2.3 Impact of Dose Factor and Gap Length 8

S2.4 Q-Factor Calculation 10

S2.4.1 Breit-Wigner-Fano Model 10

S2.4.2 Lorentzian Model 11

S3 FDTD Simulations **12**

S3.1 Distribution of the Optical Field 13

S1 Sample Fabrication

S1.1 Wafer Layout

We patterned 16 chips on an 100 mm diameter Si wafer, see Figure S1(a) and (c). Each 15 mm×15 mm chip contains a matrix 4 × 4 of metasurfaces, whose diameters are 500 µm, see Figure S1(b) and (d). On each chip we varied the electron beam lithography (EBL) exposure doses (label: 0.9-1.2) and the gap length (label: a-b). Chips labelled from 1 to 8 are realized using disconnected bow-tie antenna masks, where the mask position of the EBL defines the gap distance. This yields bow-tie antennas with nanogaps as large as ≈ 42 nm, these results are not shown in this publication. Chips from 9 to 16 have been made using connected bow-tie antenna masks, which resulted in the successful realization of sub-5 nm nanogaps.

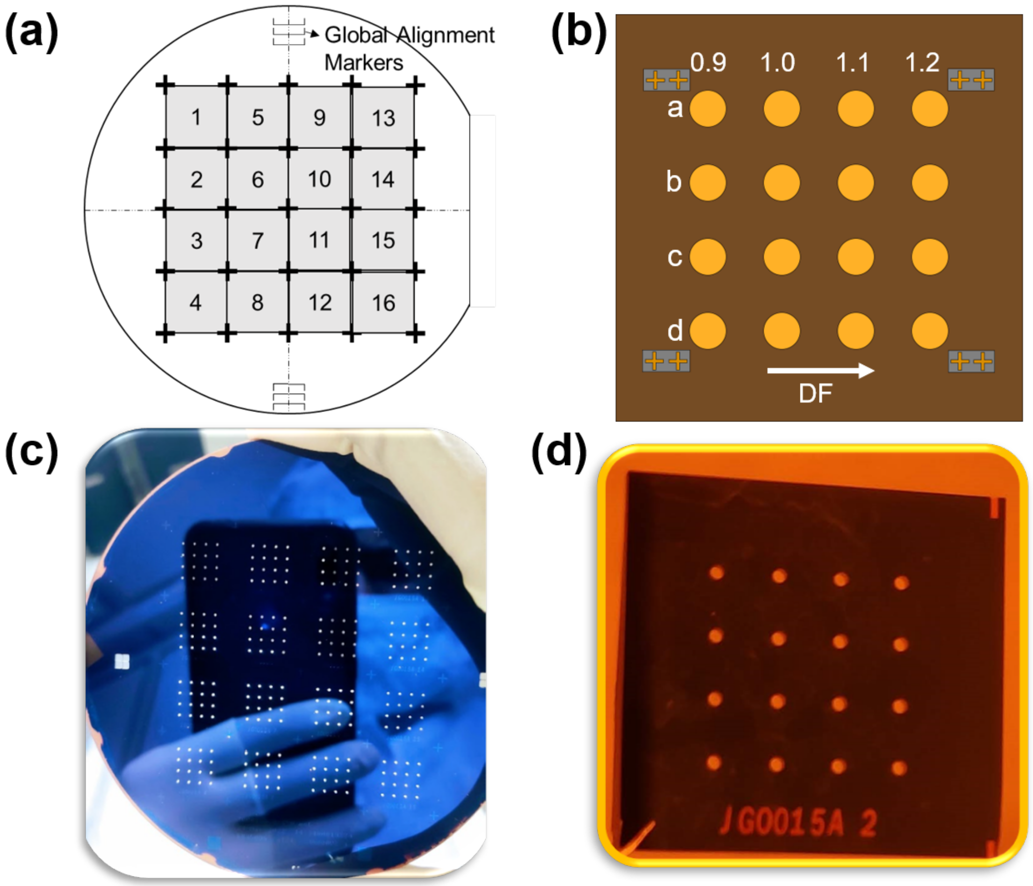

Figure S1: Wafer-scale sample design (a) showing global alignment markers and different chips on the wafer, plus markers signify wafer dicing marks. (b) A single chip layout showing the placement of local alignment markers for achieving good overlay accuracy and labels for the different metasurfaces over the chip. Figures (a) and (b) are not up to the scale and just for representation. (c) Wafer-scale sample of 100 mm diameter as fabricated and (d) 15 mm×15 mm chip containing metasurfaces over the membrane, yellow spots in 4 x 4 grid.

S1.2 Bow-tie Metasurface Design

Figure S2 represents the unit cell of the metasurfaces consisting of two gold bow-tie shaped nanoantennas displaced by 50 nm in the x-direction and 400 nm in the y-direction, and featuring a Al_2O_3 filled nanogaps in the center. The unit cell dimensions or grating period is 800 nm by 800 nm in xy-direction. The gold thickness along the z-direction (**t**) of the nanoantennas is constant and fixed at 30 nm. Table 1 reports the retrieved sizes from SEM images of the metasurfaces fabricated with different electron beam lithography (EBL) exposure doses (label: 0.9d-1.2d) and gap length (label: 1.2d-1.2b). In the paper, the BNA has a dimension as label in 1.2d. These metasurfaces are lying on a thin dielectric membrane of ALD-coated Al_2O_3 film. This membrane spans over a 500 µm diameter hole in the Si wafer and is suspended in air, providing a huge transparency range and guided mode resonances. The film has a low-stress, an ideal condition for the fabrication of thin membranes.

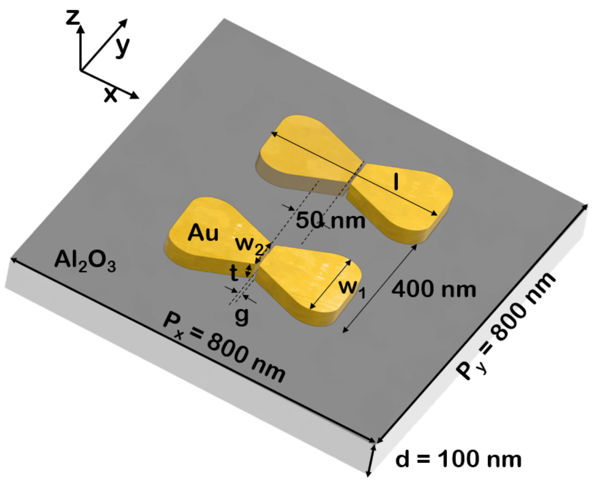

Figure S2: Designed unit cell of nanogapped metasurfaces.

**Label**	**Dose** (μC/cm^2)	w_1**-width** (nm)	w_2**-gap length** (nm)	l**-total length** (nm)
0.9d	108	123	50	275
1.0d	120	132	60	284
1.1d	132	142	65	294
1.2d	144	142	70	294
1.2c	144	139	60	292
1.2b	144	140	50	290

Table 1: Measured nanoantenna sizes from different BNA metasurfaces having different gap lengths w_2 and EBL exposure doses. All dimensions have a standard deviations below 5 nm.

S1.3 Planarization Process

To achieve vertically-oriented nanogap gratings, we planarized the samples using obliquely-angled Ar-ion beam etching. Before the etching step, we spin-coated a layer of AZ 1505 resist (MicroChemicals

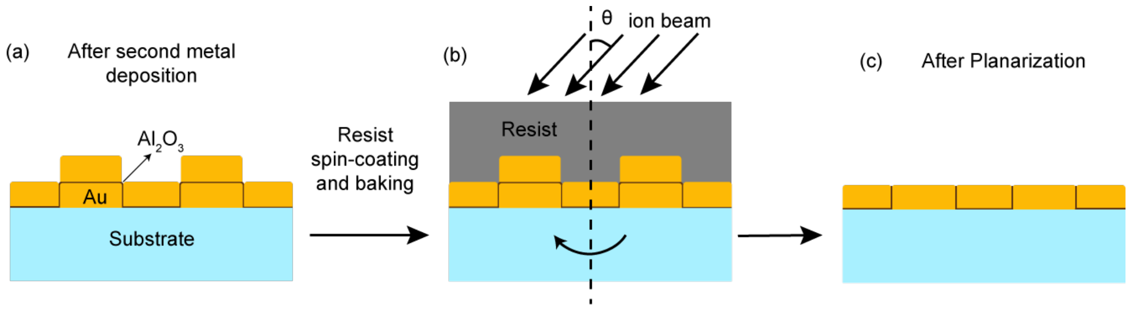

Figure S3: Planarizattion process steps: (a)-(c)A simplified schematic illustration of the planarization process based on ion beam etching.

GmbH) of 115 nm thickness and baked at 200 °C for 30 minutes. An oblique angle is chosen for the etching to compensate for the different etch rates of gold, AZ 1505 and Al_2O_3. The planarization process is optimized by first finding the etch rates at different angles for each of the materials. After that, a suitable etching angle satisfying the process requirements can be calculated and used. Figure S3 (a)-(c) demonstrates the steps involved in the planarization process.

A simplified approach to finding the etch angle (measured relative to the normal of the sample surface) involves the following formula where the time taken to etch away the left part of the dashed red box equates to the etch time required for right-part of the dashed red-box region, see Figure S4(a).

$$\frac{d_{Au}}{r_{Au}} + \frac{d_{Al_2O_3}}{r_{Al_2O_3}} = \frac{d_{AZ}}{r_{AZ}} + \frac{d_{Al_2O_3}}{r_{Au}} \tag{1}$$

where r and d represent the etch rates and thicknesses of the materials as used in the subscripts, respectively. By solving the above expression using the etch rates shown in Figure S4(b), one can find a suitable angle for etching. An operative region of etch angles identified between 55° to 65° for the different thicknesses of $d_{Al_2O_3}$ for sub-5 nm gap sizes. However, we also monitor optical emission spectroscopy (OES) signals during the etching process to prevent the over-etching. Here are the typical Ar-ion beam etching parameters that were used for the etching device (IONFAB 300, Oxford Instruments): ion energy: 400 eV, beam current: 300 mA, accelerating voltage: 400 V, rotation speed: 10 rpm, sample position: central, Ar gas flow: 8 sscm, substrate holder temperature with backside cooling: 20°C. The sample was rotated during the planarization process for the uniform etching over the wafer scale. As this planarization process is compatible with thin metal film thicknesses, here we tested with about 30 nm thick Au films, it is capable of reducing the requirements such as over-deposition of second metal than required by the nanoantennas that seems inevitable in the glancing-angle ion polishing method [1]. To process thicker metallic nanostructure, one needs to spin-coat thick resist layer to achieve planarized surface to begin with ion milling process. In the past, similar planarization processes have been utilized to reduce surface roughness and polish diamond surfaces [2].

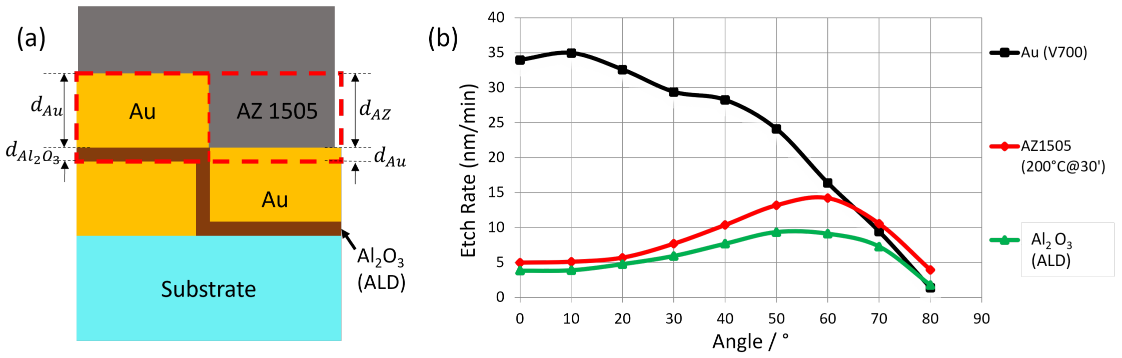

Figure S4: Suitable etch angle calculation strategy based on the obtained etch rates. (a) Schematic illustration of the side view of the nanogap structures after spin coating with the resist with the dashed red box showing the region considered for the simplified approach to calculate the etch angle. (b) Etch rates of different materials as a function of etch angle, legends show the different materials used. The angle is measured relative to the normal of the surface to be etched.

S1.4 Thin Film Membrane Fabrication

For transmission measurements, we chose to fabricate nano-gap bow-tie antennas on a thin film of Al_2O_3 suspended in air to avoid strong absorption in the silicon substrate. In addition, the thin film supports a fundamental guided mode showing a sharp resonance around 825 nm, which helps in the excitation of the surface waves at the Rayleigh condition. However, this membrane is not necessary to fabricate sub-5 nm plasmonic metasurfaces. At the beginning of fabrication, a 100 nm thick ALD-coated Al_2O_3 film on Si is deposited on each wafer. To realize the membranes, we employed photolithography in combination with mask-aligner lithography and subsequent Si dry-etching against the patterned mask using inductive coupled plasma (SENTECH- SI-591). The Si dry etching is performed on the backside of the bow-ties, after a protective resist is coated on the antennas. Since we had a good etch ratio of 60:1 between Si and Al_2O_3, no additional etch stop layer was required. After the membrane fabrication, we removed the protective resist coating by soaking the sample in acetone for 30 minutes and rinse in IPA. Unfortunately, the removal process of the resist coating produced residuals of materials sticking on the backside of the membrane. Nonetheless, the effect of these residuals on the plasmonic resonances are minimum given they are located on the backside of the membrane, on the opposite side of the antennas.

S2 Linear Characterization

S2.1 Linear Transmission Setup

Figure S5 shows the experimental setup used to measure the linear transmission of the 0th diffraction order versus the input wavelength. As broadband light source a supercontinuum laser (SuperK Com-

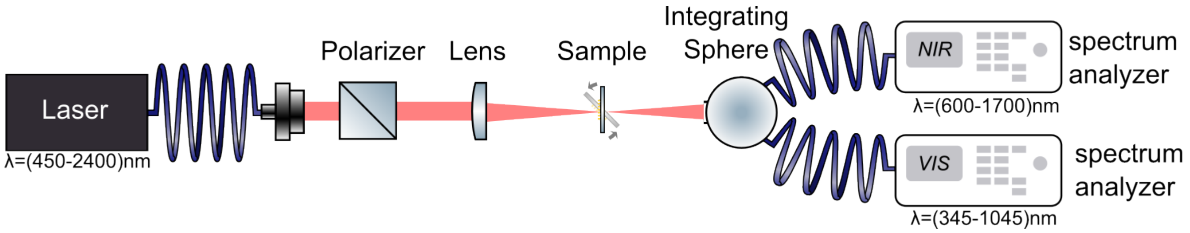

Figure S5: Schematic of the characterization setup.

pact by NKT, λ = 450 nm-2400 nm) is used, passing through a polarizer (GT10-A by Thorlabs) to fix a defined linear polarization. The spatial coherent beam is weakly focused with a 300 mm lens to a theoretical beam diameter 2ω of 190 µm to 260 µm on the sample, diameter increases from λ=500 nm to λ=1700 nm due to chromatic aberration. The 0th diffraction order is coupled into a polymer integrating sphere (by Artifex). The 20 mm diameter integrating sphere has two fiber output ports, thus allowing the simultaneous use of two fiber based spectrometers working in different spectral ranges. The sphere also strongly suppresses the spatial coherence of the output beam, so that no interference effects arise in the multi-mode fibers and making the fiber coupling largely insensitive to the alignment.

For the visible (VIS) range we use a compact spectrometer (USB4000 by Ocean Optics, λ = 345 nm-1045 nm), whereas for the near infrared (NIR) range we employed a scanning spectrometer (AQ6370D by Yokogawa, λ = 600 nm-1700 nm). The optical resolution of the NIR spectrometer is 2 nm and the sampling resolution is 0.4 nm. For the VIS spectrometer the sampling resolution is ≈0.19 nm. Both spectra are later combined into the shown spectrum. There is a spectral overlap between both spectrometers, the values from the VIS spectrometer are taken for wavelengths shorter than 900 nm due to the better signal-to-noise ratio. Finally, the measured spectra are normalized with respect to a reference spectrum measured in the absence of any sample.

S2.2 Homogeneity of Fabrication

We confirm the homogeneity of the nanogap metasurface by measuring the transmission spectrum at different chips and metasurface positions. In Figure S6, the measured responses show a similar response at different positions on the same metasurface using the 300 mm focusing lens. The BNA design therefore hardly changes on the 500 µm metasurface. This measurement was carried out for different chips coming from different locations on the wafer (see Fig. S1a), which should have the same fabrication parameters. We observed a slight change of the LSPR response due to changes of the gap position offset and membrane thickness. In Figure S7, we used a 75 mm lens for further decreasing of the spot size to 48 µm to 65 µm The measured responses of the metasurface with 3 nm nanogaps is also similar, this confirms the homogeneity of the optical response also for small probe beam sizes.

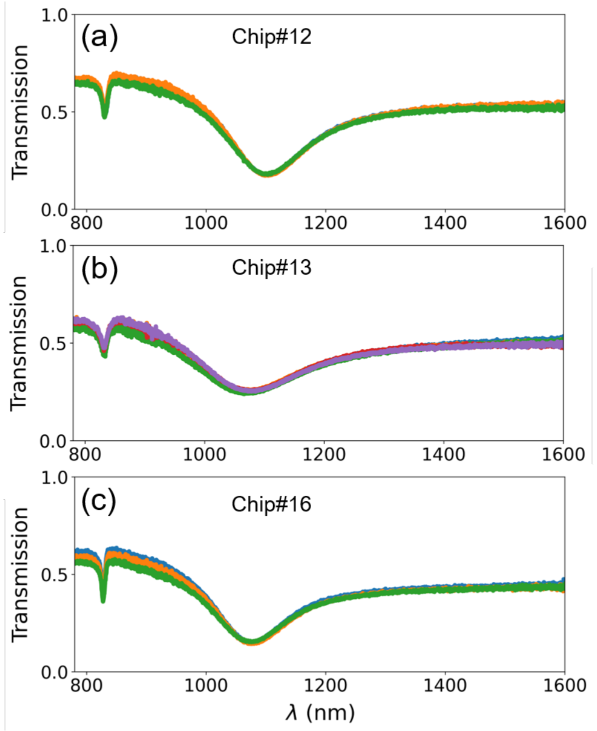

Figure S6: Measured 0th order transmission spectra from different chips of 2 nm nanogap metasurfaces, BNA dimension corresponding to 1.2d label. The different positions of the probe beam with a focusing lens of 300 mm. Dimensions of BNA should be the same. Each curve corresponds to different spots (three in (a), five in (b), and three in (c)) on the metastructure.

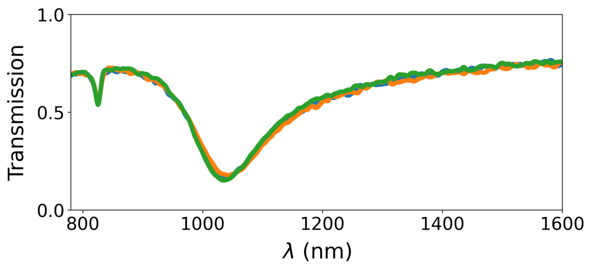

Figure S7: Measured 0th order transmission spectra of a 3 nm nanogap metasurface at different positions of the probe beam on the membrane at normal incidence. Each curve corresponds to 3 different spots on the metastructure.

S2.3 Impact of Dose Factor and Gap Length

We investigated the impact of the fabrication dose factors of the EBL exposure on the optical response. Higher dose factors, lead to overall larger BNA dimensions (see Tab. 1, label: 0.9d-1.2d), while the gap distance is constant. The dose factor modifies the Q factor of the CR and LSPR resonances (Fig. S8), which emphasizes the importance of selective optimized exposure parameters for obtaining desired optical properties of plasmonic devices.

We also varied the gap length ω_2 while remaining other BNA dimensions (see Tab. 1, label: 1.2d-1.2b). Decreasing the gap length w_2 of BNA redshifts the LSPR position for a gap size of 2 nm strongly

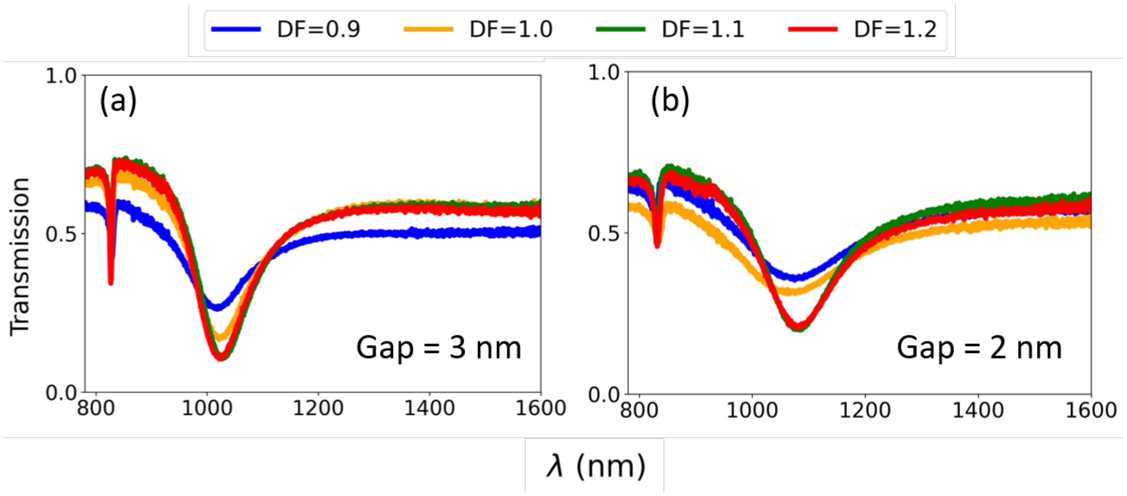

Figure S8: Measured 0th order transmission spectra from different metasurfaces of varying EBL exposure dose factor in legends, a base dose of $120\,\mu C/(cm)^2$ was used, on a single chip of 15 mm x 15 mm for the gap sizes of (a) 3 nm and (b) 2 nm.

(see Fig. S9) and changes the Q factor of the LSPR.

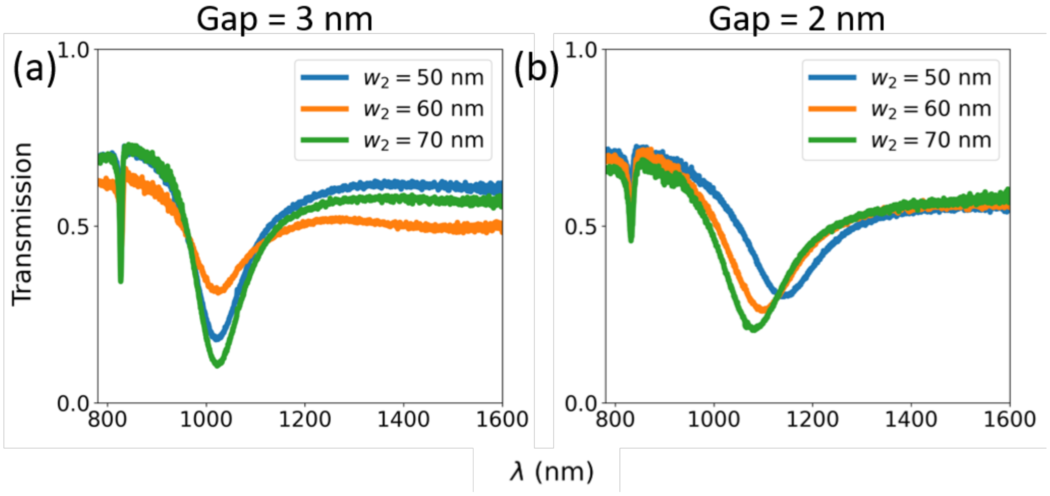

Figure S9: Measured 0th order transmission spectra from different metasurfaces of varying gap length (w_2), in legends, for the gap sizes of (a) 3 nm and (b) 2 nm.

S2.4 Q-Factor Calculation

In correspondence to each resonance, FWHM and Q-factor of both CR and LSPR were determined by fitting the experimental results with a combination of Lorentzian (for LSPR), Breit-Wigner[3] (based on Breit-Wigner-Fano function for CR) and Exponential (based on exponential decay function to account for decaying transmission) models, specifically using the lmfit python library[4]. The Q-factor is calculated using the following definition

$$Q = \frac{\lambda_0}{\Delta\lambda}. \tag{2}$$

We summarized the calculated Q-factor values, obtained from simulation and experiments for 2 nm and 3 nm nanogap samples, in Table 2, exhibiting good agreement.

Gap (nm)	Experiment			Simulation		
	$Q_{\text{CR, Fano}}$	$Q_{\text{CR, Lorentz}}$	$Q_{\text{LSPR, Lorentz}}$	$Q_{\text{CR, Fano}}$	$Q_{\text{CR, Lorentz}}$	$Q_{\text{LSPR, Lorentz}}$
3	167	165	8.0	356	363	6.9
2	139	138	7.2	318	323	6.6

Table 2: Q-factors of metasurfaces calculated from the extracted FWHM values by fitting the acquired transmission spectra and simulated responses.

In correspondence to CR resonance, we fitted transmission spectra using the Fano and Lorentzian models as described below to extract full-width half-maximum (FWHM) and calculate the quality factor (Q-factor).

S2.4.1 Breit-Wigner-Fano Model

The Fano model of transmission fit is represented by the following equation:

$$T = T_0 + A\frac{(q+\Omega)^2}{1+\Omega^2} \tag{3}$$

where:

- T_0 is transmission offset,
- A is transmission amplitude,
- q is Fano parameter, indicating the asymmetry of the line shape,
- Ω is a dimensionless parameter defined as $\Omega = \frac{(\lambda-\lambda_0)}{\sigma/2}$, where λ, λ_0, and σ correspond to wavelength, resonance wavelength, and FWHM, respectively.

S2.4.2 Lorentzian Model

The Lorentzian model of transmission fit is expressed as:

$$T = T_0 + A\frac{1}{1+\Omega^2} \tag{4}$$

Parameters T_0, A, Ω, λ, λ_0, and σ have the same meanings as in the Fano model.

S3 FDTD Simulations

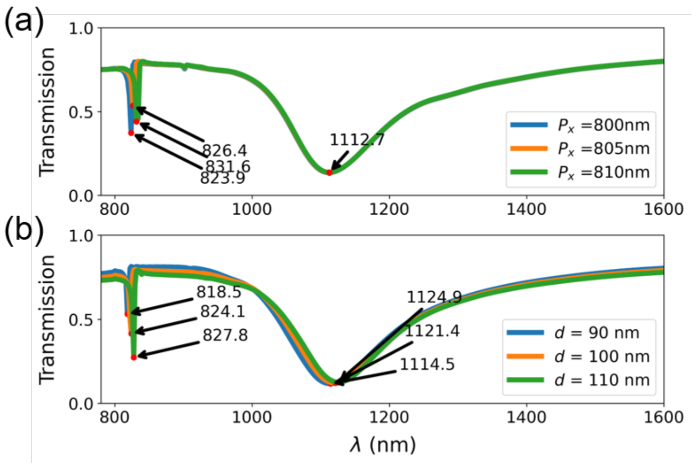

Figure S10: Simulated transmission spectra of the metasurfaces for (a) different periods and (b) the membrane thicknesses, d, as reported in the label for the case of a 3 nm gap.

Figure S10 shows the simulated transmission spectrum when the period along the x-direction is changed in steps of 5 nm and when the membrane thickness, d, is varied in steps of 10 nm. These simulations are carried out to show that small variations in the period are potentially caused by the stress in the membrane and/or as a result of slightly different membrane thickness from the designed value of $d = 100$ nm. The results showing the shifts of the CR position hint that offset from the defined period can affect CR resonance position, as observed in our experiments.

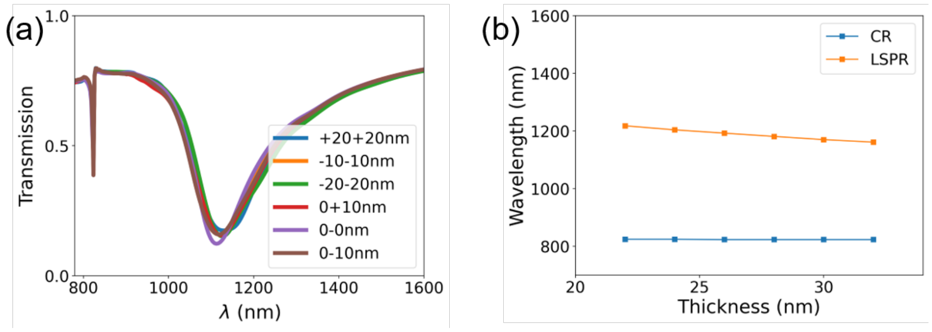

Figure S11: (a) Simulated transmission spectra of 3 nm nanogap metasurfaces having positional offsets of maximum 20 nm from the center of BNA. Each nanogap position of the two Bow-Ties is independently varied in steps of 10 nm, displayed in the legend. (b) LSPR and CR positions plotted as a function of BNA metasurface thickness (**t**) for 2 nm gap case, the corresponding resonances are indicated in legends.

The effect of the nanogap offset from the center is discussed in Figure S11(a). Due to symmetrical reasons, positive and negative offsets provide the same transmission spectra. In this case, the offset only affects the LSPR, with the resonance moving at maximum 15 nm. The other factor influencing

LSPR positions is the BNA thickness ($\mathbf{t}$) variation of across the wafer. In Figure S11(b), the simulated response demonstrate characteristically the LSPR variation by change of 4 nm in the nanoantenna thickness induces roughly 20 nm shift in LSPR.

S3.1 Distribution of the Optical Field

Figure S12 shows the field distribution when a monochromatic plane wave is impinging perpendicularly on the metasurface. The cross-section taken at the interface of membrane and the nanoantenna across the transverse plane xy is pictured. Two wavelengths corresponding to the CR (824 nm) and LSPR (1156 nm) are plotted. In correspondence to the CR, i.e. in the presence of a Rayleigh-Wood anomaly, the field spreads over the metasurfaces. In the case of LSPR, fields remain extremely localized around the nanoantennas. Regardless of the wavelength, the field is mainly concentrated inside the nanogap with a similar peak in two cases.

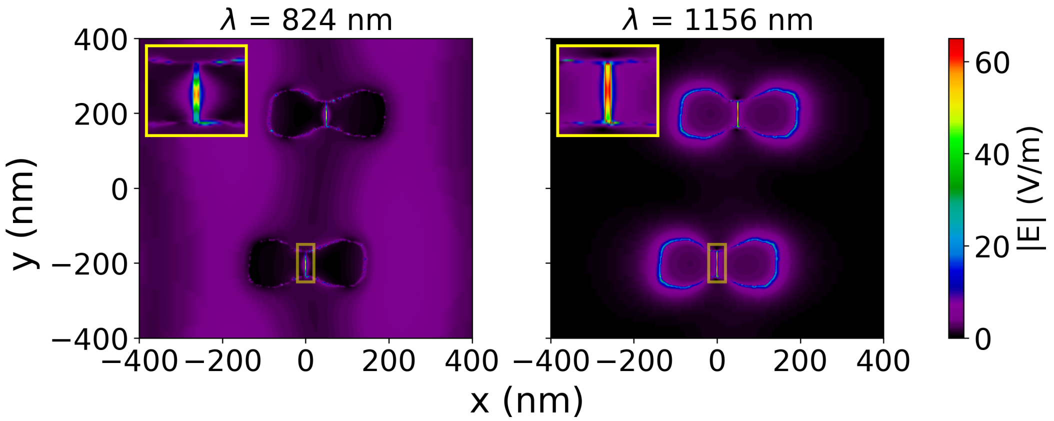

Figure S12: Simulated optical field in a 2 nm nanogap BNA metasurface on the plane xy. The field is sampled at the interface of the membrane and the nanoantennas.

References

[1] D. Yoo, N.-C. Nguyen, L. Martin-Moreno, *et al.*, "High-throughput fabrication of resonant metamaterials with ultrasmall coaxial apertures via atomic layer lithography," *Nano letters*, vol. 16, no. 3, pp. 2040–2046, 2016.

[2] Y. Li, H. Takino, and F. Frost, "Ion beam planarization of diamond turned surfaces with various roughness profiles," *Opt. Express*, vol. 25, no. 7, pp. 7828–7838, Apr. 2017.

[3] U. Fano, "Effects of configuration interaction on intensities and phase shifts," *Physical review*, vol. 124, no. 6, p. 1866, 1961.

[4] M. Newville, T. Stensitzki, D. B. Allen, M. Rawlik, A. Ingargiola, and A. Nelson, "Lmfit: Nonlinear least-square minimization and curve-fitting for python," *Astrophysics Source Code Library*, ascl–1606, 2016.
